# Zingerone Modulates Neuronal Voltage-Gated Na^+^ and L-Type Ca^2+^ Currents

**DOI:** 10.3390/ijms23063123

**Published:** 2022-03-14

**Authors:** Ming-Chi Lai, Sheng-Nan Wu, Chin-Wei Huang

**Affiliations:** 1 Chi-Mei Medical Center, Department of Pediatrics, Tainan 71004, Taiwan; vickylai621@gmail.com; 2 Department of Physiology, College of Medicine, National Cheng Kung University, Tainan 70101, Taiwan; 3 Institute of Basic Medical Sciences, Medical College, National Cheng Kung University, Tainan 70101, Taiwan; 4 Department of Neurology, College of Medicine, National Cheng Kung University, Tainan 70101, Taiwan

**Keywords:** zingerone (gingerone; vanillylacetone), voltage-gated Na^+^ current, persistent Na^+^ current, L-type Ca^2+^ current, hysteresis

## Abstract

Zingerone (ZO), a nontoxic methoxyphenol, has been demonstrated to exert various important biological effects. However, its action on varying types of ionic currents and how they concert in neuronal cells remain incompletely understood. With the aid of patch clamp technology, we investigated the effects of ZO on the amplitude, gating, and hysteresis of plasmalemmal ionic currents from both pituitary tumor (GH_3_) cells and hippocampal (mHippoE-14) neurons. The exposure of the GH_3_ cells to ZO differentially diminished the peak and late components of the *I*_Na_. Using a double ramp pulse, the amplitude of the *I*_Na(P)_ was measured, and the appearance of a hysteresis loop was observed. Moreover, ZO reversed the tefluthrin-mediated augmentation of the hysteretic strength of the *I*_Na(P)_ and led to a reduction in the *I*_Ca,L_. As a double ramp pulse was applied, two types of voltage-dependent hysteresis loops were identified in the *I*_Ca,L_, and the replacement with BaCl_2_-attenuated hysteresis of the *I*_Ca,L_ enhanced the *I*_Ca,L_ amplitude along with the current amplitude (i.e., the *I*_Ba_). The hysteretic magnitude of the *I*_Ca,L_ activated by the double pulse was attenuated by ZO. The peak and late *I*_Na_ in the hippocampal mHippoE-14 neurons was also differentially inhibited by ZO. In addition to acting on the production of reactive oxygen species, ZO produced effects on multiple ionic currents demonstrated herein that, considered together, may significantly impact the functional activities of neuronal cells.

## 1. Introduction

Zingerone (ZO, gingerone, vanillylacetone), a nontoxic methoxyphenol isolated from the rhizome of ginger (*Zingiber officinale* Roscoe), has been used as a flavor additive in spiced oils and in perfumery to introduce exotic aromas. It is widely recognized to have potent anti-inflammatory, antidiabetic, antilipolytic, antidiarrheal, antispasmodic, and anti-tumor properties [1,2,3,4]. ZO has also been reported to be particularly efficient at scavenging free radicals and reactive oxygen species in the body, in addition to inhibiting the enzymes involved in the generation of these reactive oxygen species [1,5]. Finally, it has recently been demonstrated to induce the production of reactive oxygen species linked to ZO-induced apoptotic changes in colon cancer cells [6].

It should be noted that ZO can perturb some types of membrane ionic currents in electrically excitable cells. For instance, ZO has been reported to activate the transient receptor potential ankyrin-1 (TRPA1) and transient receptor potential vanilloid-1 (TRPV1) ion channels in spinal substantia gelatinosa neurons and trigeminal ganglion neurons [7,8,9]. Earlier studies have also demonstrated its effectiveness both in inhibiting the pacemaker potentials of interstitial cells of Cajal via NO/cGMP-dependent ATP-sensitive K^+^ (K_ATP_) channels [10] and in modulating the amplitude of voltage-gated K^+^ currents in prostate cancer cells. The latter effect is assumed to be linked to the anti-neoplastic effect of ZO [11]. Moreover, a very recent study has reported that ZO attenuates status epilepticus by blocking hippocampal neurodegeneration via the regulation of redox imbalance, inflammation, and apoptosis [5]. Finally, it has also been shown that ZO potentially inhibits colonic motility in rats [12,13], and ginger extracts ameliorate both deltamethrin-induced testicular abnormalities and cypermethrin- or lambda-cyhalothrin-induced thyroid disorders in rats [2,14,15,16]. Therefore, it is important to further study whether ZO produces any specific effects on neuronal ion channels.

Voltage-gated Na^+^ (Na_V_) channels, which constitute the whole-cell voltage-gated Na^+^ current (*I*_Na_), are widely known to participate in the initiation and propagation of action potentials in various excitable cells. Nine Na_V_ channel α-subunits (Na_V_1.1–1.9) are functionally expressed in mammalian tissues, including the endocrine system, the central and peripheral nervous systems, the skeletal muscles, and the heart [17,18,19]. The mRNA transcripts for the α-subunits Na_V_1.1, Na_V_1.2, Na_V_1.3, and Na_V_1.6 have been identified in GH_3_ cells [18]. An earlier study has shown the effectiveness of eugenol (4-allyl-2-methoxyphenol), another essential oil extracted from cloves, in differentially modulating the magnitude of peak and late Na^+^ currents [20], while tefluthrin (Tef), a type-I pyrethroid, has been identified as an activator of *I*_Na_ [21,22]. The voltage-dependent hysteresis inherently present in Na_V_ channels may potentially significantly perturb the electrical behavior in cells, resulting in either an overload of Na^+^ owing to an excessive influx of Na^+^ or hormonal secretions in various types of excitable cells, especially during exposure to pyrethroid insecticides [2,16]. Therefore, it would be interesting to determine whether cell exposure to ZO is capable of decreasing the hysteresis loops present at both the high- and low-threshold voltages of the current activated by the double (i.e., isosceles-triangular) ramp voltage.

The L-type Ca^2+^ channel, known as the dihydropyridine receptor, is part of the family of voltage-dependent Ca^2+^ (Ca_V_) channels. “L” stands for long-lasting, referring to the length of activation, and the L-type Ca^2+^ current (*I*_Ca,L_) is assumed to be carried through this channel, which has four subunits: Ca_V_1.1 (CACNA1S), Ca_V_1.2 (CACNA1C), Ca_V_1.3 (CACNA1D), and Ca_V_1.4 (CACNA1F). Several pore-forming subunits of Ca_V_ channels (i.e., Ca_V_1.1, Ca_V_1.2, Ca_V_1.3, and Ca_V_3.1) have been identified in GH_3_ cells [18]. The regulatory function of these channels located in endocrine cells (e.g., pituitary cells) is based on an influx of Ca^2+^ in response to transient depolarization, where the channels serve as intracellular messengers controlling a variety of functions of the pituitary cells [23,24,25]. To date, however, the extent to which ZO impacts this type of Ca^2+^ current (i.e., *I*_Ca,L_) is unclear.

Voltage-gated K^+^ (K_V_) channels also perturb membrane excitability, and these currents are ubiquitous in neuroendocrine or hippocampal neurons. K_V_3.1-K_V_3.2 channels have been shown to be the major determinants of the delayed-rectifier K^+^ current (*I*_K(DR)_) in pituitary GH_3_ cells [25,26]. In addition, the cation current through a hyperpolarization-activated cation current (*I*_h_) elicits membrane depolarization toward a threshold, generating an action potential and reducing membrane resistance and the magnitude of excitatory and inhibitory postsynaptic potentials. These currents play a major role in controlling neuronal excitability, the dendritic integration of synaptic potentials, synaptic transmission, and rhythmic oscillatory activity in individual neurons and neuronal networks [27]. However, whether the presence of ZO influences the amplitude and gating of *I*_K(DR)_ is largely unknown.

Therefore, in light of the considerations outlined above, this work is an attempt to address the question of whether ZO has a perturbing effect on different types of ionic currents, including *I*_Na_, persistent Na^+^ currents (*I*_Na(P)_), *I*_Ca,L_, *I*_h_ and *I*_K(DR)_, through the membranes of excitable cells present in pituitary GH_3_ cells. The *I*_Na_ in mouse hippocampal mHippoE-14 neurons was also evaluated during cell exposure to ZO.

## 2. Results

### 2.1. Effect of ZO on the Voltage-Gated Na^+^ Current (I_Na_) Measured in GH_3_ Cells

**Figure 1:**
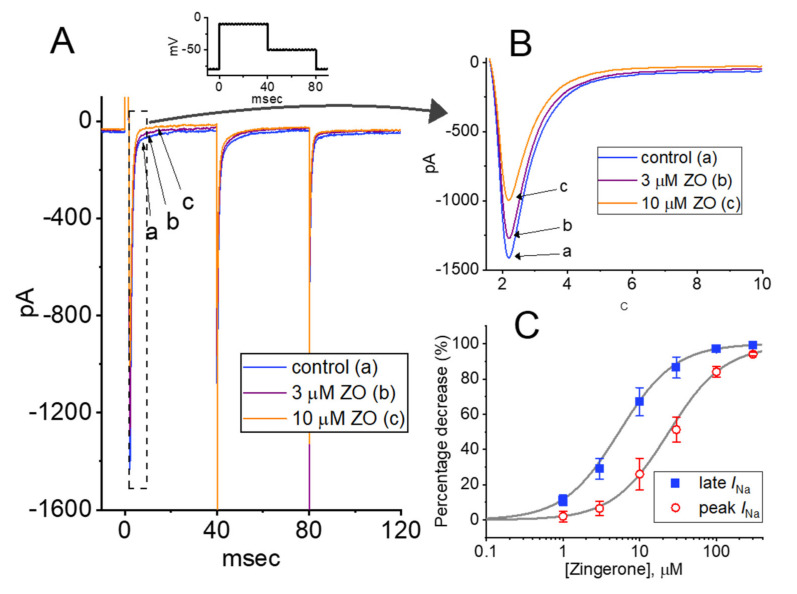
Effect of zingerone (ZO) on voltage-gated Na^+^ current (*I*_Na_) recorded from pituitary tumor (GH_3_) cells. This set of measurements was undertaken in cells bathed in Ca^2+^-free Tyrode’s solution in which 10 mM TEA was present, where the recording pipette was backfilled with a Cs^+^-containing solution. (**A**) Representative current traces obtained in (a) the control situation (i.e., ZO was not present) and during cell exposure to 3 μM ZO (b) or 10 μM ZO (c). Inset is the applied voltage pulse protocol. (**B**) Expanded records from the dashed box in (**A**). (**C**) Concentration-dependent inhibition of ZO on the peak (○) and late (■) amplitude of *I*_Na_ measured from GH_3_ cells (mean ± SEM; *n* = 7 for each point). Peak or late amplitude with or without ZO addition, respectively, taken at the beginning or end of a rapid depolarizing pulse ranging from −80 to −10 mV. Solid smooth lines are fits to the modified Hill equation (as elaborated in the Section 4).

In the first stage of the experiments, we explored whether the presence of ZO produced any perturbations of the amplitude or gating of the *I*_Na_ as a result of rapid membrane depolarization in these cells. We kept the GH_3_ cells immersed in Ca^2+^-free Tyrode’s solution containing 10 mM TEA and 0.5 mM CdCl_2_, and the recording pipette was backfilled with Cs^+^-containing solution (the compositions of the solutions will be stated later). As the whole-cell configuration proceeded, we voltage-clamped the examined cells at −80 mV and thereafter applied a rapid depolarizing pulse to −10 mV for a duration of 40 msec to induce a *I*_Na_. As has been observed in various cell types, including pituitary cells, the *I*_Na_ during abrupt depolarization was clearly manifested in the form of an inward current, along with rapid activation and inactivation time courses [21,25,28]. Importantly, as the cells were continually exposed to different concentrations of ZO, the peak and late amplitudes of the *I*_Na_ activated by 40 msec depolarizing pulses ranging from −80 to −10 mV progressively decreased (Figure 1A,B). For example, the addition of 10 μM of ZO decreased the amplitude of the peak and late *I*_Na_ in a time-dependent manner to 993 ± 47 pA (*n* = 7; paired, t(6) = 3.072, *p* = 0.014) and 87 ± 9 pA (*n* = 7; paired, t(6) = 3.211, *p* = 0.012), respectively, from the control values of 1308 ± 88 pA (*n* = 7) and 198 ± 19 pA (*n* = 7), respectively. After the agent was removed, the amplitude of the current returned to 1289 ± 82 pA (*n* = 7; paired, t(6) = 3.18, *p* = 0.012) and 192 ± 17 pA (*n* = 7; paired, t(6) = 3.311, *p* = 0.011), respectively, indicating that the action of the ZO is readily reversible. The slow component of the inactivation time constant (τ_inact(S)_) of the *I*_Na_ also appeared to be reduced, as demonstrated by a measurable decrease from 12.6 ± 3.4 msec to 7.2 ± 1.1 msec (*n* = 7; paired, t(6) = 2.452, *p* = 0.03) in the presence of 10 μM of ZO. On the other hand, the fast component remained unchanged in the presence of 10 μM of ZO, with a control value of 1.9 ± 0.4 msec and 2.0 ± 0.3 msec in the presence of 10 μM ZO (*n* = 7, paired, t(6) = 1.921, *p* = 0.07). Furthermore, the sigmoidal concentration-dependent inhibitory effect of ZO on the amplitude of the *I*_Na_ (peak and late components) measured at the start and end (from −80 mV to −10 mV) of a 40 msec depolarization of the command voltage was produced, as illustrated in Figure 1C. The effective IC_50_ value required to produce the ZO-mediated inhibition of peak and late *I*_Na_ in GH_3_ cells was estimated to be 23.7 and 5.4 μM, respectively (Figure 1C). It is thus reasonable to assume that the exposure of cells to ZO differentially inhibits peak and late *I*_Na_ activated in response to a brief depolarizing pulse.

### 2.2. Enhanced Amplitude and Hysteresis by Tefluthrin (Tef) of Persistent Na^+^ Current (I_Na(P)_) Reversed by the Addition of ZO

**Figure 2:**
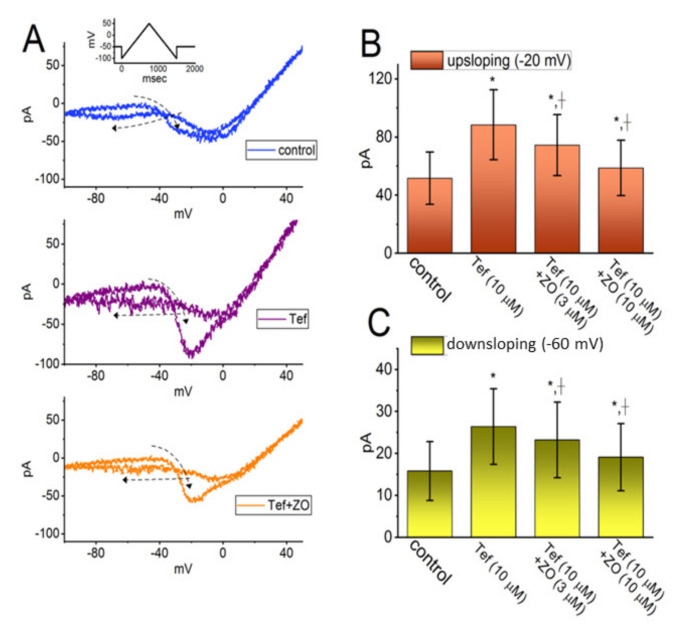
Inhibitory effect of ZO on Tef-mediated augmentation in persistent *I*_Na_ (*I*_Na(P)_) activated by a double ramp pulse in GH_3_ cells. In this set of whole-cell current recordings, we held the potential applied to the examined cell at −50 mV, and a triangular ramp voltage with a duration of 1.5 s (i.e., a ramp speed of ±0.2 mV/msec) was applied to elicit *I*_Na(P)_. That is, the whole-cell currents were evoked in response to the forward (ascending from −100 to +50 mV) and backward (descending from +50 to −100 mV) ramp voltage-clamp command. (**A**) Representative current traces obtained in the control period (upper) and during exposure to Tef (10 μM) (middle) or to Tef (10 μM) plus ZO (10 μM). The uppermost inset is the applied pulse protocol, while the broken arrows in each panel are the direction of the current trajectory over time. The figure-eight pattern in the voltage-dependent hysteresis of *I*_Na(P)_ elicited by double ramp voltage with a duration of 1.5 s (or ramp speed of ±0.2 mV/msec) should be noted. Panels (**B**,**C**), respectively, show the effects of Tef (10 μM) and Tef (10 μM) plus ZO (3 or 10 μM) on the *I*_Na(P)_ amplitude activated by the upsloping (ascending) and downsloping (descending) limb of a 1.5 s triangular ramp pulse (mean ± SEM; *n* = 7 for each bar). The current amplitude in (**B**) or (**C**) was taken at either the −20 mV (i.e., high-threshold *I*_Na(P)_) or at −60 mV (i.e., low-threshold *I*_Na(P)_), respectively. * Significantly different from controls (*p* < 0.05) and † significantly different from the 10 μM Tef alone group (*p* < 0.05). Panel (A): (One-way ANOVA, F(4,30) = 3.693, *p* = 0.01) and panel (B): (One-way ANOVA, F(4,30) = 3.822, *p* = 0.01).

Ginger extract has previously been reported to attenuate testicular abnormalities induced by deltamethrin as well as thyroid disorders induced by cypermethrin or lambda-cyhalothrin. Both deltamethrin and cypermethrin are pyrethroid insecticides that are structurally similar to tefluthrin (Tef). We therefore proceeded to investigate whether exposing cells to ZO had a moderating effect on the Tef-induced augmentation of *I*_Na(P)_ evoked by a double ramp pulse in GH_3_ cells. In the whole-cell voltage-clamp recordings, the examined cell was voltage-clamped at −50 mV, and a set of double ramp pulses ranging between −100 and +50 mV at a rate of 0.05 Hz was applied to it using digital-to-analog conversion (Figure 2A). As shown in previous studies [22,28,29], when the cells were exposed to 10 μM of Tef alone, the amplitude of the *I*_Na(P)_ at both the high- and low-threshold voltages was activated in response to a robust increase in the upsloping (forward, ascending) and downsloping (backward, descending) limbs, respectively, of the upright triangular ramp voltage. In addition, a striking figure-eight (i.e., ∞) hysteresis loop appeared in the instantaneous current vs. voltage *I–V* relationship of *I*_Na(P)_ activated by the ramp pulse (Figure 2A). The data suggested a dynamic voltage dependence in *I*_Na(P)_ [29,30].

When the upright double ramp pulse was applied to the cell for 1.5 s (or ramp speed of ±0.2 mV/msec) in the presence of 10 μM of Tef, the peak *I*_Na(P)_ measured at the levels of −20 mV (i.e., the high-threshold *I*_Na(P)_) and −60 mV (i.e., the low-threshold *I*_Na(P)_) during the ascending and descending ends of the triangular ramp pulse was increased to 88.5 ± 23.9 and 26.4 ± 9.0 pA (*n* = 7; paired, t(6) = 2.651, *p* = 0.02), respectively, from the control values (measured at an isopotential level) of 51.8 ± 18.1 pA (*n* = 7; paired, t(6) = 2.732, *p* = 0.02) and 15.8 ± 6.8 pA (*n* = 7; paired, t(6) = 2.734, *p* = 0.02), respectively. It should be noted, as demonstrated in Figure 2B,C, that the subsequent addition of 10 μM of ZO, in the continued presence of 10 μM of Tef, led to a progressive decrease in the amplitude of both the high- and low-threshold *I*_Na(P)_ in response to double ramp pulses to 58.8 ± 18.8 pA (*n* = 7; paired, t(6) = 2.812, *p* = 0.02) and 19.1 ± 8.1 pA (*n* = 7; paired, t(6) = 2.833, *p* = 0.02), respectively. It is conceivable, therefore, that the introduction of ZO, while retaining Tef, is capable of reducing the strength of the voltage-dependent hysteresis observed in the instantaneous *I–V* relationship of *I*_Na(P)_ responding to a double ramp voltage in GH_3_ cells (Figure 2).

### 2.3. Effect of ZO on the L-Type Ca^2+^ Current (I_Ca,L_) in GH_3_ Cells

**Figure 3:**
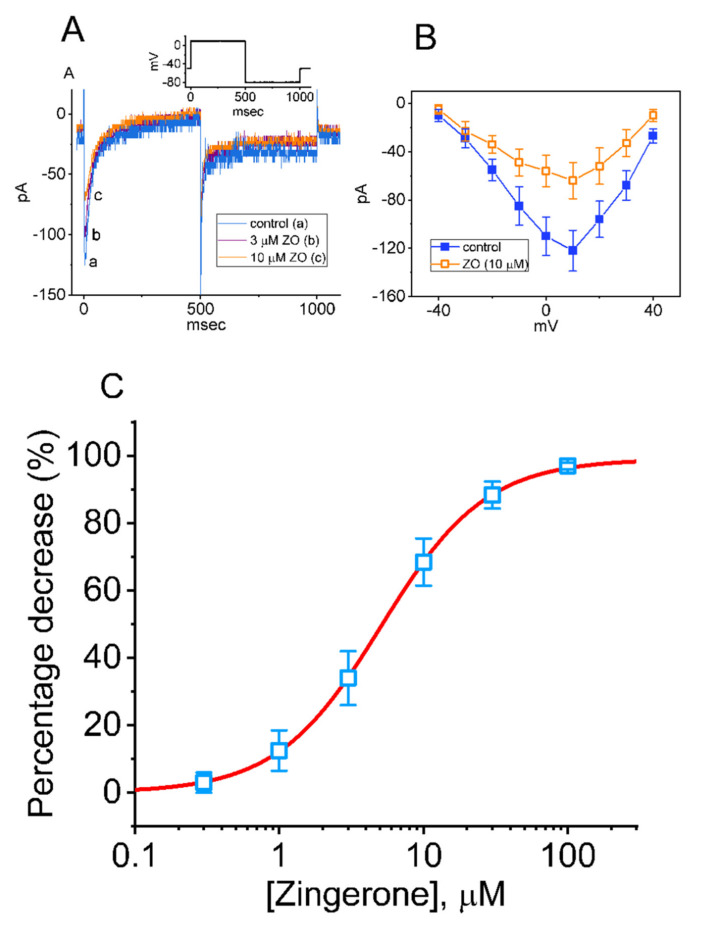
Inhibitory effect of ZO on the L-type Ca^2+^ current (*I*_Ca,L_) identified in GH_3_ cells. In these experiments, the cells were kept immersed in a normal Tyrode’s solution containing 1.8 mM CaCl_2_. The recording electrode was filled with Cs^+^-containing solution. (**A**) Representative current traces obtained under (a) the control situation (i.e., ZO was not present), and in the presence of 3 μM ZO (b) or 10 μM ZO (c). The inset shows the applied voltage-clamp protocol. (**B**) Mean current vs. voltage (*I–V*) relationships of peak *I*_Ca,L_ in the absence (■) and presence (□) of 10 μM ZO (mean ± SEM; *n* = 7 for each point). The current amplitude was measured at the start of each membrane depolarization to voltages ranging between −40 and +40 mV from a holding potential of −50 mV. (**C**) Concentration-dependent effect of ZO on the amplitude (□) of *I*_Ca,L_ evoked by membrane depolarization to +10 mV from a holding potential of −50 mV (mean ± SEM; *n* = 8 for each point). The current amplitude was measured at the start of the depolarizing pulse during exposure to various concentrations of ZO. The continuous smooth line indicates the goodness-of-fit to the modified Hill equation, as stated in the Section 4.

In another set of experiments, we investigated the effect of ZO on another type of inward current (i.e., *I*_Ca,L_). We kept cells immersed in a normal HEPES-buffered Tyrode’s solution in which 1.8 mM of CaCl_2_, 10 mM of TEA, and 1 μM of TTX were present, and the electrode was backfilled with a Cs^+^-containing solution. As shown in Figure 3A, as the cells were continuously exposed to different concentrations of ZO, the peak amplitude of *I*_Ca,L_ in response to a 500 msec membrane depolarization ranging from −50 to +10 mV progressively decreased. The overall *I–V* relationship of the peak amplitude of *I*_Ca,L_ with and without the addition of 10 μM of ZO is illustrated in Figure 3B, with Figure 3C plotting the relationship between the ZO concentration and the percentage decrease in peak *I*_Ca,L_. Following the application of least-squares fitting and the Hill model, the half-maximal concentration of IC_50_ required for ZO to have an inhibitory effect on the *I*_Ca,L_ was found to be 9.1 μM. The Hill coefficient was estimated at around 1. At a concentration of 100 μM, ZO almost fully eliminated the peak amplitude of *I*_Ca,L_. These experimental results indicate that ZO has an inhibitory effect on *I*_Ca,L_ in GH_3_ cells. However, neither the activation nor the inactivation time course of peak *I*_Ca,L_ in response to a rectangular depolarization pulse ranging from −50 to +10 mV was altered during exposure to ZO. Additionally, no change (e.g., the threshold and peak potentials activated by membrane depolarization) in the overall *I–V* relationship of peak *I*_Ca,L_ was shown in the presence of ZO.

### 2.4. Biophysical Properties of Voltage-Dependent Hysteresis of I_Ca,L_ Activated by a Double Ramp Pulse

**Figure 4:**
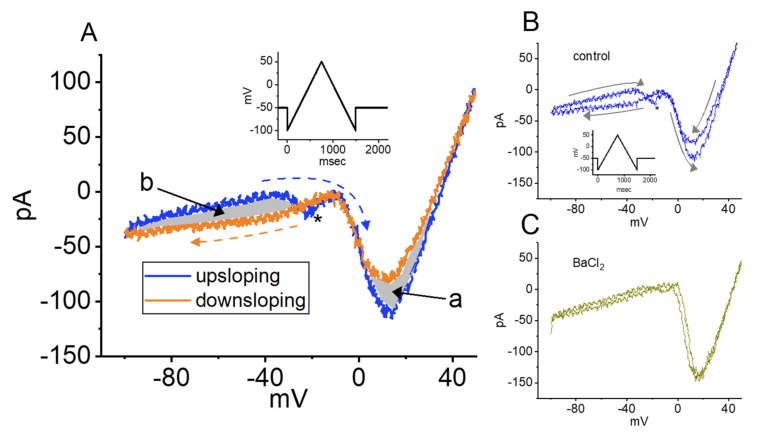
Characterization of voltage-dependent hysteresis (i.e., an instantaneous current–voltage relationship) of *I*_Ca,L_ identified in GH_3_ cells. Cells were bathed in a normal Tyrode’s solution containing 10 mM TEA and 1 μM TTX, and the electrode was filled with a solution containing Cs^+^. (**A**) Current traces were evoked by a 1.5 s double ramp voltage (as indicated in the inset). The ascending limb is shown in blue, and the descending one is shown in orange. The arrow denotes the direction of the current trajectory over time, while the asterisk notes the appearance of *I*_Na(P)_ inhibited by replacement with BaCl_2_. The grey area labeled as a and b, respectively, illustrates the hysteretic loop of *I*_Ca,L_ (i.e., high- and low-threshold loops). (**B**,**C**) show the hysteretic loop of the current trace obtained in the control period (i.e., *I*_Ca,L_) and the BaCl_2_ substitution (i.e., Ba^2+^ inward current [*I*_Ba_]), respectively. The representative current trace in (**B**) is the control (i.e., ZO was not present), while that in (**C**) was obtained when 2 mM BaCl_2_ was substituted for CaCl_2_. It should be noted that the hysteretic strength (i.e., both loops) of *I*_Ca,L_ elicited by the double ramp pulse (indicated in the inset of (**B**)) was diminished as the replacement of CaCl_2_ with BaCl_2_ was made.

We next examined whether voltage-dependent hysteresis of the *I*_Ca,L_ occurred when activated by a double ramp pulse and whether the presence of ZO modified the hysteretic strength of the current. In these experiments, when whole-cell current recordings were made, we held the examined cell in a voltage clamp at −50 mV and then applied a 1.5 s upright isosceles-triangular ramp pulse ranging between −100 and +50 mV with a ramp speed of 0.2 mV/msec in order to obtain measurements of the characteristics of the hysteretic behavior (Figure 4A). Under these conditions, the amplitudes of *I*_Ca,L_ activated at the upsloping (ascending) and downsloping (descending) ends of the triangular ramp voltage were clearly distinguishable. There appeared to be two voltage-dependent hysteresis loops, one a high-threshold anticlockwise loop and the other a low-threshold clockwise loop, of the current elicited by the double ramp pulse (Figure 4A). When BaCl_2_ (2 mM) was replaced with CaCl_2_ in normal Tyrode’s solution (i.e., cells were perfused with normal Tyrode’s solution containing 1.8 mM of CaCl_2_, and then perfused with Ca^2+^-free Tyrode’s solution containing 2 mM of BaCl_2_), the slope following the peak of the low-threshold component was less steep than that observed in the control phase (i.e., in normal Tyrode’s solution). It seems that the low-threshold component inactivated rather quickly, which might be in part due to the higher driving force of Ca^2+^. As shown in Figure 4B,C, the amplitude of the Ba^2+^ inward current (*I*_Ba_) should have increased as Ca^2+^-dependent inactivation was eliminated. Additionally, there was a displacement of the limbs of the ramp voltage around 0 mV, indicating the occurrence of voltage-dependent hysteresis (Figure 4C). Thus, changing the voltage dependence by a couple of mV could have a great impact on the activity [31]. In this regard, the hysteretic strength of the current noticeably diminished, although the amplitude of the *I*_Ba_ was increased (Figure 4B,C).

### 2.5. Effect of ZO, Nimodipine (Nimo), and BaCl_2_ Replacement on the Hysteresis of I_Ca,L_

**Figure 5:**
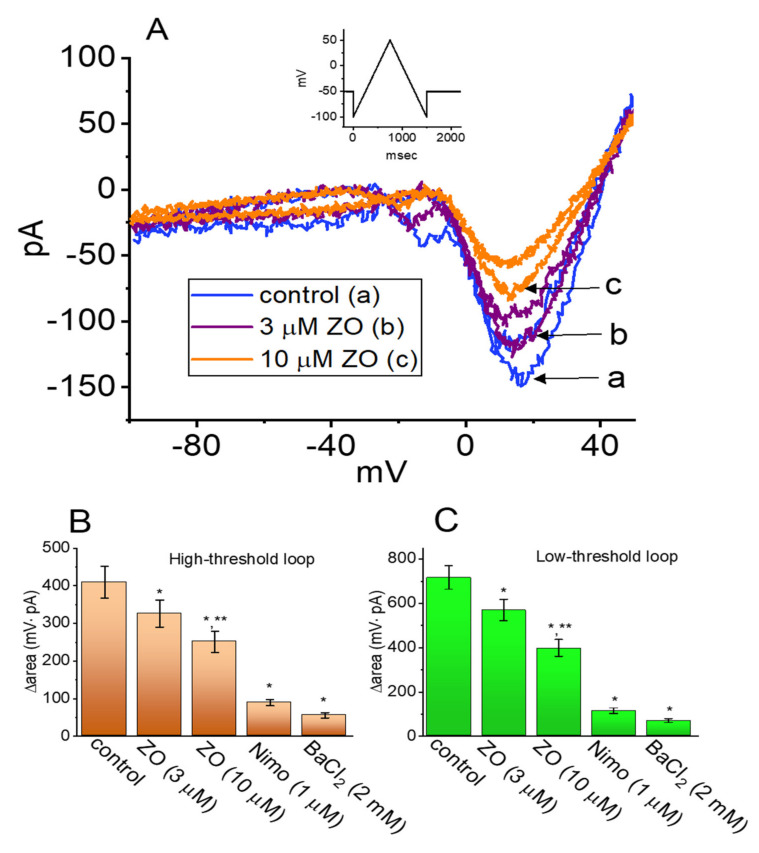
Inhibitory effect of ZO on *I*_Ca,L_ activated by a double ramp pulse in GH_3_ cells. (**A**) Representative current traces obtained in the control period (a) and in the presence of 3 μM ZO (b) or 10 μM ZO (c). The inset in the upper part illustrates the voltage protocol. (**B**,**C**), respectively, illustrate the effects of ZO (3 and 10 μM) or BaCl_2_ replacement on the hysteretic area (high- and low-threshold loop) of *I*_Ca,L_ (mean ± SEM; *n* = 7 for each bar). * Significantly different from the controls (*p* < 0.05) and ** significantly different from the ZO (3 μM)-alone groups (*p* < 0.05). Panel (**A**): (One-way ANOVA, F(4,30) = 4.156, *p* = 0.01) and panel (**B**): (One-way ANOVA, F(4,30) = 3.692, *p* = 0.01).

The effects of ZO, Nimodipine (Nimo), and BaCl_2_ replacement on the voltage-dependent hysteresis of the *I*_Ca,L_ induced in response to an upright double ramp voltage in GH_3_ cells were investigated. As shown in Figure 5A–C, when the cells were continually exposed to ZO, the hysteresis of the current was progressively and robustly decreased. The degree of the voltage-dependent hysteresis of the *I*_Ca,L_ was determined on the basis of the difference in the areas (Δarea) (as indicated by the grey area in Figure 4A) under the curves in the forward and backward limbs of the isosceles-triangular ramp voltage. For example, the addition of 10 μM of ZO resulted in an evident reduction in Δarea for the high- and low-threshold hysteresis loops, from 409 ± 42 mV·pA (*n* = 7; paired, t(6) = 2.923, *p* = 0.02) and 715 ± 53 mV·pA (*n* = 7; paired, t(6) = 3.012, *p* = 0.02) to 251 ± 28 mV·pA (*n* = 7; paired, t(6) = 3.015, *p* = 0.02) and 396 ± 38 mV·pA (*n* = 7; paired, t(6) = 3.011, *p* = 0.02), respectively. Conversely, with the addition of Nimo (1 μM) and Ba^2+^ (2 mM) as a replacement for the external Ca^2+^ (as the charge carrier through the Ca^2+^ channels), the Δarea for the double ramp-induced current was measurably decreased in GH_3_ cells (Figure 5B,C). Therefore, Nimo was viewed as an effective inhibitor of the *I*_Ca,L_ [23,24].

### 2.6. Limited Inhibition of ZO on the Hyperpolarization-Activated Cation Current (I_h_) in GH_3_ Cells

**Figure 6:**
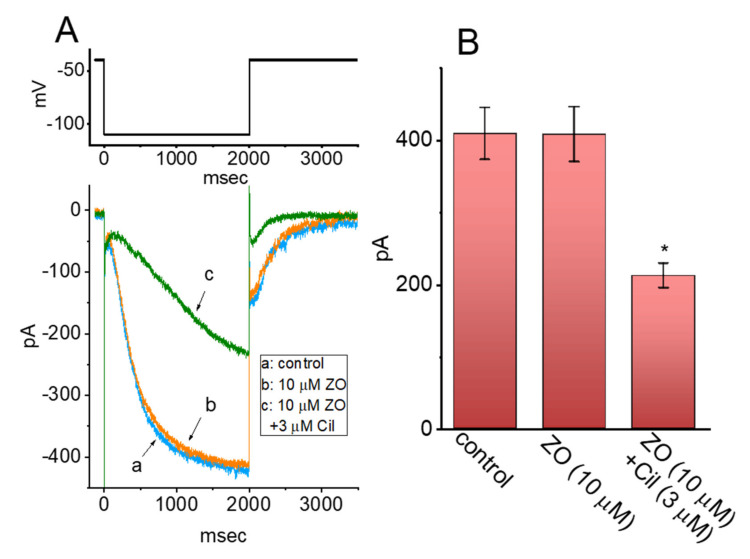
Inability of ZO to perturb the hyperpolarization-activated cation current (*I*_h_) in GH_3_ cells. The experiments were conducted in cells bathed in a Ca^2+^-free Tyrode’s solution, with the internal solution backfilled with a K^+^-containing solution. (**A**) Representative current trace obtained in the control period (a) and cell exposure to 10 μM ZO (b) or 10 μM ZO plus 3 μM cilobradine (Cil) (c). The upper part is the applied voltage-clamp protocol. (**B**) Summary bar graph showing the effects of ZO or ZO plus cilobradine (Cil) on *I*_h_ amplitude (mean ± SEM; *n* = 7 for each bar). The current amplitude was obtained at the endpoint of a 2 s hyperpolarizing pulse ranging from −40 to −110 mV. * Significantly different from the control or the 10 μM ZO-alone group (*p* < 0.05). (One-way ANOVA, F(2,18) = 5.085, *p* = 0.02).

Previous investigations have shown the effectiveness of ZO in perturbing the pacemaker potential found in interstitial cells of Cajal isolated from the small intestine [10]. In line with these findings, we examined whether the amplitude of the Hyperpolarization-Activated Cation Current (*I*_h_) inherently existing in GH_3_ cells was subject to being modified by ZO. The whole-cell experiments were undertaken with cells bathed in a Ca^2+^-free Tyrode’s solution containing 1 μM of TTX, after which the electrode was filled using a K^+^-containing solution. As the examined cells were hyperpolarized from −40 to −110 mV for 2 s to induce an *I*_h_, the presence of 10 μM of ZO failed to alter the amplitude and gating of the *I*_h_ (Figure 6). However, we observed that, in the continued presence of 10 μM of ZO, the addition of 3 μM of cilobradine (Cil) to the bath effectively decreased the amplitude of the long *I*_h_, as well as increasing the activation time constant of the current. It has been suggested that Cil is an inhibitor of the *I*_h_ [7].

### 2.7. Mild Inhibitory Effect of ZO on the Delayed-Rectifier K^+^ Current (I_K(DR)_) in GH_3_ Cells

**Figure 7:**
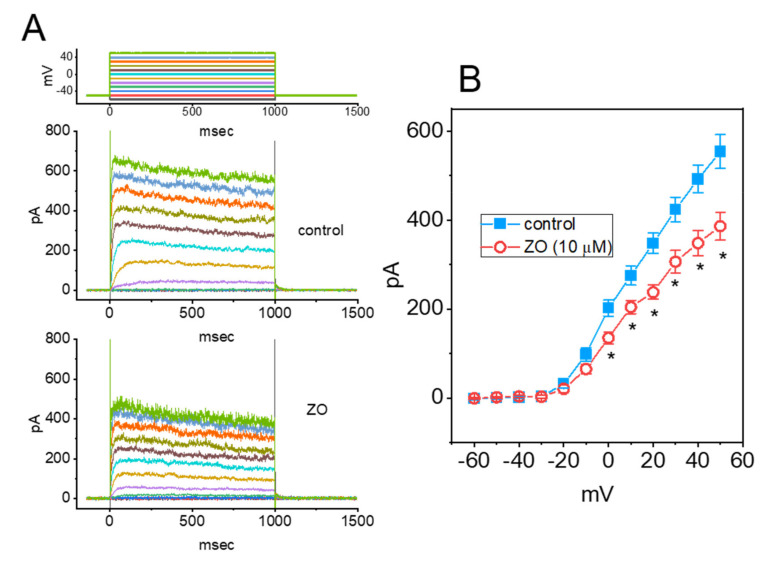
Mild inhibition of a Delayed-Rectified K^+^ Current (*I*_K(DR)_) caused by ZO in GH_3_ cells. In these experiments, we kept cells bathed in Ca^2+^-free Tyrode’s solution, and the recording pipette was filled with K^+^-containing solution. As the whole-cell configuration was commenced, we voltage-clamped the examined cell at −50 mV and applied various voltage pulses ranging between −60 and +50 mV at 10 mV. (**A**) Representative current traces obtained in the control period (upper) and after application of 10 μM OZ (lower). The uppermost part indicates the applied voltage-clamp protocol. (**B**) The mean *I–V* relationship of *I*_K(DR)_ taken without (■) or with (○) the addition of 10 μM ZO (mean ± SEM; *n* = 8 for each point). The current amplitude was taken at the endpoint of each voltage command. * Significantly different from the controls taken at the same potential (*p* < 0.05).

Previous studies have reported the ability of ZO to modulate the amplitude of the Delayed-Rectifier K^+^ Current (*I*_K(DR)_) present in prostate neoplastic cells [11]. We thus examined whether the application of ZO modifies the amplitude or gating of the *I*_K(DR)_ in such cells. The cells were bathed in a Ca^2+^-free Tyrode’s solution in order to prevent interference by either Ca^2+^-activated K^+^ currents or voltage-gated Ca^2+^ currents, and the recording pipette was backfilled with a K^+^-containing solution. As shown in Figure 7A,B, when the cells were exposed to 10 μM of ZO, the amplitude of the *I*_K(DR)_ was slightly decreased; however, no change in either the activation or the inactivation of the current was detected in its presence. Figure 7B illustrates the mean *I–V* relationship of the current obtained in the absence and presence of 10 μM of ZO. As compared to its inhibitory effect on *I*_Na_ and *I*_Ca,L_, ZO was less likely to block the *I*_K(DR)_ identified in GH_3_ cells, despite its ability to inhibit the amplitude of the voltage-gated K^+^ current in prostate cancer cells [11].

### 2.8. Effect of ZO on I_Na_ in Mouse Hippocampal (mHippoE-14) Neurons

**Figure 8:**
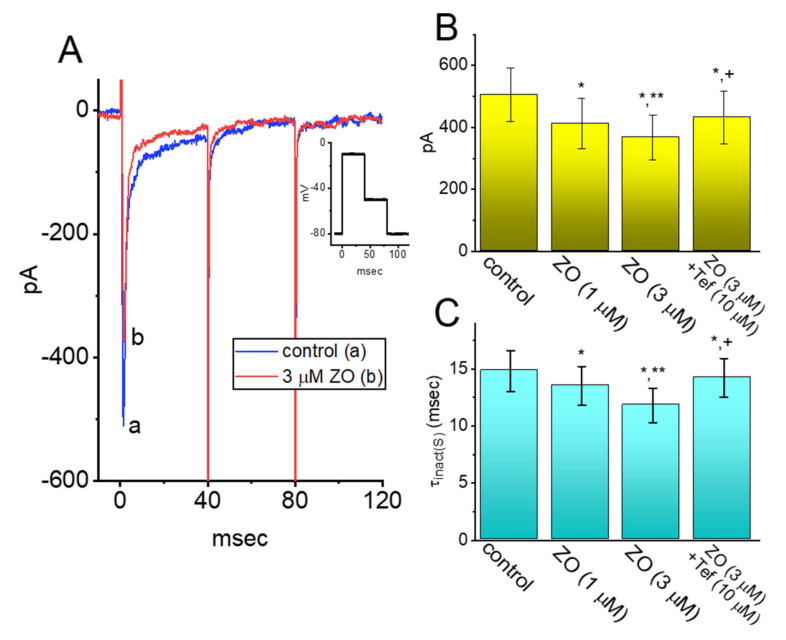
Inhibitory effect of ZO on *I*_Na_ on mHippoE-14 hippocampal neurons. (**A**) Representative current traces obtained in the absence (a) or presence of 1 μM ZO (b). The inset shows the pulse protocol used in this study. Panel (**B**,**C**) are summary bar graphs demonstrating the effects of ZO and ZO plus Tef on the peak amplitude or τ_inact(S)_ of depolarization-activated *I*_Na_, respectively (mean ± SEM; *n* = 7 for each bar). * Significantly different from the control (*p* < 0.05), ** significantly different from the 1 μM ZO-alone group (*p* < 0.05), and + significantly different from the 3 μM ZO-alone group (*p* < 0.05). Panel (**A**): (One-way ANOVA, F(3,24) = 3.351, *p* = 0.03) and panel (**B**): (One-way ANOVA, F(3,24) = 4.012, *p* = 0.02).

Previous studies have reported the benefits of ZO in age-related neurological disorders [8,32]. In a final set of experiments, we explored whether the amplitude and gating of the *I*_Na_ in another type of excitable cells (hippocampal neurons) were perturbed by the presence of ZO. The cells were bathed in Ca^2+^-free Tyrode’s solution containing 10 mM of TEA, and we filled up the electrode with a Cs^+^-containing solution. As demonstrated in Figure 8, within 1 min of exposing the cells to ZO, the amplitude of the peak *I*_Na_ was obviously decreased in combination with the shortened inactivation time constant of the current elicited by a brief depolarizing pulse ranging from −80 to −10 mV. For example, the application of 3 μM of ZO significantly and consistently decreased the peak *I*_Na_ from 505 ± 86 pA (*n* = 7; paired, t(6) = 3.212, *p* = 0.02) to 368 ± 73 pA (*n* = 7; paired, t(6) = 3.089, *p* = 0.01), and also decreased the τ_inact(S)_ value from 14.8 ± 1.8 msec (*n* = 7; paired, t(6) = 2.892, *p* = 0.02) to 11.8 ± 1.5 msec (*n* = 7; paired, t(6) = 2.889. *p* = 0.02). No change in the fast component of the inactivation time constant of the *I*_Na_ was seen in the presence of 3 μM of ZO (2.3 ± 0.4 msec for the control vs. 2.4 ± 0.5 msec with ZO; *n* = 7, paired, t(6) = 1.653, *p* = 0.10). Moreover, the addition of 10 μM of Tef, while keeping the 3 μM of ZO, increased the peak *I*_Na_ to 432 ± 85 pA and the τ_inact(S)_ value to 14.2 ± 1.7 msec. The IC_50_ values required for the ZO-mediated inhibition of the peak and late *I*_Na_ were estimated to be 23.7 and 5.4 μM, respectively. Consistent with previous observations made with GH_3_ cells, the *I*_Na_ in mHippoE-14 neurons is subject to inhibition by ZO.

## 3. Discussion

In the present study, as GH_3_ cells were exposed to ZO, the peak and late amplitudes of the *I*_Na_ were differentially inhibited. The addition of Tef, a pyrethoid, was observed to activate the *I*_Na_ and to slow down its inactivation time course [21,22,29]. A voltage sensor was energetically coupled to the Na_V_-channel activation in response to a double ramp pulse and appears to be a conformationally flexible region of the channel protein. It is possible that the voltage-dependent movement of the S4 segment in the Na_V_ channels was overly perturbed, consequently leading to the enhancement of the coupling of the pore domain to the voltage-sensing domain. Therefore, during the exposure of the cells to Tef, a mode shift in voltage sensitivity to the gating charge movements may have emerged, which was dependent on the previous state (or conformation) of the channel [28,29,30,33]. This unique type of voltage-dependent hysteresis inherently present in Na_V_ channels may play a significant role in the perturbance of electrical behavior, the overloading of Na^+^ owing to an excessive Na^+^ influx, and the secretion of hormones in various types of excitable cells, especially during exposure to pyrethroid insecticides [2,16]. Furthermore, it must be kept in mind that the subsequent application of ZO, while retaining Tef, produced a measurable reduction in the hysteretic strength of the *I*_Na(P)_ elicited in response to the double ramp voltage.

In this study, upon the application of an abrupt double ramp voltage, a hysteresis loop with a figure-eight pattern eliciting the *I*_Ca,L_ was also detected. The trajectory of the current induced by the ramp pulse protocol revealed two loops, a high-threshold anticlockwise loop and a low-threshold clockwise loop, during hysteresis. As extracellular Ca^2+^ ions were replaced with Ba^2+^ ions, the low-threshold current at the downsloping end of the triangular ramp diminished, whereas the high-threshold current at the downsloping end of the ramp increased. The formation of a low-threshold clockwise loop was likely brought about by the magnitude of the Ca^2+^-activated nonselective cationic currents or the late component of the *I*_Ca,L_ [23,34]. As a result, the replacement of Ca^2+^ ions with Ba^2+^ ions increased the amplitude of the *I*_Ca,L_ (i.e., the *I*_Ba_), whereas the voltage-dependent hysteresis of the current activated by the double ramp pulse was aberrantly reduced. More importantly, cell exposure to ZO decreased the area of both the high- and low-threshold hysteresis loops of the current activated by the ramp voltage.

In earlier studies on the pharmacokinetic effects of the oral administration of ZO, the half-maximal inhibitory concentration (IC_50_) values of a self-microemulsion drug delivery system that either contained ZO or was free of ZO was reported to be 8.45 μg/mL and 13.3 μg/mL (43.5 μM and 68.5 μM), respectively [35]. These values are considerably higher than the IC_50_ values for the ZO-mediated inhibition of the *I*_Na_ (peak and late components = 23.7 μM and 5.4 μM) and the *I*_Ca,L_ (9.1 μM) observed in this study. Moreover, the hysteretic strength of the *I*_Na(P)_ and *I*_Ca,L_ induced by the double ramp voltage was clearly reduced in the presence of ZO. The findings of this study suggest that the effects of ZO on ionic currents are pharmacologically and even therapeutically relevant, on the condition that in vivo findings similar to the present observations can be produced.

In our experimental work, the internal solution used for the whole-cell recordings contained ATP at a concentration of 3 mM, which is known to cause the complete suppression of K_ATP_-channel activity [36]. In contrast to previous observations [10], the subsequent addition of diazoxide, an activator of K_ATP_ channels [36], was not observed to attenuate the ZO-mediated inhibition of the *I*_K(DR)_ in GH_3_ cells. We also observed that ZO was ineffective at modifying the amplitude and gating of the *I*_h_ in response to hyperpolarization sustained during a long period. Therefore, the inhibition of K^+^ currents produced by ZO in GH_3_ cells appears to be independent of the direct interaction between ZO and the activity of the K_ATP_ and HCNx channels, despite the functional expression of these channels in pituitary cells [7,25,36,37].

Previous studies have revealed the ability of ZO to modify the magnitude of transient receptor potential (TRP) channels (e.g., TRPA1 and TRPV1) [9,34,38]. Therefore, it seems likely that the TRP superfamily of cation channels in GH_3_ cells [18] would be modified by the presence of ZO. However, in contrast to those of the *I*_Na_ and *I*_Ca,L_, the biophysical properties of TRP-mediated currents are relatively time- or voltage-independent [9,18,34], and this type of current has been linked to the absence of voltage-gated activation, of an inactivation and deactivation time course, and of voltage-dependent hysteresis. It is therefore unlikely that the *I*_Na_ and *I*_Ca,L_ inhibited by ZO mainly arise from the alteration of the activity of TRP channels by ZO. Moreover, the subsequent application of reduced glutathione (GSH, 10 mM) and superoxide dismutase (SOD, 500 U/mL) failed to modify ZO-induced changes in the amplitude of the *I*_Na_ and *I*_Ca,L_ (data not shown), which makes it unlikely that there is a direct link between the inhibitory action of ZO on ionic currents and its antioxidative properties [1,5,6,14]. However, the production of reactive oxygen species was not measured in this study. The extent to which ZO-mediated perturbations of ionic currents lead to changes in reactive oxygen species remains to be determined.

A very recent study has reported that ZO ameliorates the adverse effects of status epilepticus by blocking hippocampal neurodegeneration via the regulation of redox imbalance, inflammation, and apoptosis [5]. The blocking of the *I*_Na_ and *I*_Ca,L_ evidenced in the present study supports the potential of ZO to attenuate seizure activity [38,39,40]. The question of whether ZO can serve as an important agent to counteract epileptogenesis and other neuronal hyperexcitability disorders deserves further investigation. The possibility that ZO is superior to barbiturates and benzodiazepines for in vivo approaches to anti-epileptic management needs to be further studied in in vivo studies. As an inhibitor of the *I*_Na_ and *I*_Ca,L_, the benefit and toxicity of ZO need to be carefully investigated.

## 4. Materials and Methods

### 4.1. The Chemicals and Solutions Used in This Work

Zingerone (ZO, gingerone, vanillylacetone, 4-(4-hydroxy-3-methoxyphenyl)-2-butanone, 4-(3-methoxy-4-hydroxyphenyl)-butan-2, C_11_H_14_O_3_, IUPAC name: 4-(4-hydroxy-3-methoxyphenyl)butan-2-one), diazoxide, nimodipine (Nimo), reduced glutathione (GSH), superoxide dismutase, tefluthrin (Tef), tetraethylammonium chloride (TEA), and tetrodotoxin (TTX) were produced by Sigma-Aldrich (Merck Ltd., Taipei, Taiwan). For the stock solutions, all drugs were dissolved in distilled water or dimethylsulfoxide (DMSO) and stored at −20 °C, after which they were diluted in an extracellular solution to reach the final concentrations. The final concentration of DMSO in the bath solution was always <0.1%. For the cell preparation, all culture media, horse serum, fetal bovine or calf serum, L-glutamine, and trypsin/EDTA were from HyClone^TM^ (Thermo Fischer, Kaohsiung, Taiwan), unless stated otherwise. All other chemicals, including CsCl_2_, CsOH, BaCl_2_, EGTA, aspartic acid, and HEPES, were of laboratory grade and obtained from standard sources. In the experiments reported in this work, we used twice-distilled water that was de-ionized through a Millipore-Q system (Merck, Darmstadt, Germany).

The extracellular solution for the GH_3_ cells (i.e., a normal HEPES-buffered Tyrode’s solution) used in this work was composed as follows: 136.5 mM of NaCl, 5.5 mM of KCl, 1.8 mM of CaCl_2_, 0.53 mM of MgCl_2_, 5.5 mM of MgCl_2_, and 5.5 mM of HEPES adjusted to a pH of 7.4 with NaOH. To measure the whole-cell *I*_h_ and *I*_K(DR)_, we backfilled the patch electrode with a solution composed as follows: 140 mM of KCl, 1 mM of MgCl_2_, 3 mM of Na_2_ATP, 0.1 mM of Na_2_GTP, 0.1 mM of EGTA, and 5 mM of HEPES adjusted to a pH of 7.2 by adding KOH. In the experiments measuring the *I*_Na_ or *I*_Ca,L_ currents, the KCl in the pipette solution was replaced with an equimolar concentration of CsCl in order to block K^+^ currents, and the pH was adjusted to 7.2 by adding CsOH. The solution was filtered using a sterile syringe filter with a 0.22 μm pore size (Bio-Check, New Taipei City, Taiwan).

### 4.2. Cell Preparations

GH_3_ pituitary cells were acquired from the Bioresources Collection and Research Center (BCRC-60015; Hsinchu, Taiwan), whereas the embryonic mouse hippocampal cell line (mHippoE-14; CLU198) was obtained from CELLutions Biosystems (Cedarlane^®^; Burlington, ON, Canada). The GH_3_ cells were maintained and subcultured in Ham’s F-12 media supplemented with 15% horse serum (*v/v*), 2.5% fetal calf serum (*v/v*), and 2 mM of L-glutamine [41,42], and the mHippoE-14 cells were maintained in Dulbecco’s modified Eagle’s medium supplemented with 10% fetal bovine serum (*v/v*) and 2 mM of L-glutamine [43]. Under our experimental conditions, the cell viability remained at 80–90% for at least two weeks. The cells were maintained at 37 °C in a humidified environment of 5% CO_2_ in a 95% air incubator. Trypsin/EDTA (0.05%, HyClone^TM^) was used for trypsinization. The measurements were undertaken five or six days after the cells were cultured (60–80% confluence). The GH_3_ cells used in this study were cultured with the passage range of 15–27.

### 4.3. Electrophysiological Measurements with Data Recordings and Analyses

Ic50 measurements were made; cells grown to confluence were harvested and rapidly transferred to a homemade recording chamber firmly mounted on the stage of an inverted Olympus fluorescent microscope (CKX-41; Yuan Yu, Taipei City, Taiwan). We placed the cells in an extracellular solution (i.e., a normal HEPES-buffered Tyrode’s solution) at room temperature (22 to 25 °C). After the cells were left to adhere to the bottom of the chamber for several minutes, the recordings were carried out. The patch electrodes were pulled from a Kimax-51 capillary with an outer diameter of 1.5–1.8 mm (#34500; Kimble; Dogger, New Taipei City, Taiwan) using either a P-97 horizontal puller (Flaming/Brown, Sutter, Novato, CA, USA) or a PP-830 vertical puller (Narishige; Major Instruments, Tainan, Taiwan). The recording pipettes used in the experiments had a tip diameter of ~1 μm, and after being fire-polished, they had resistances of 3–5 MΩ when filled with the various internal solutions described above. They were mounted in an air-tight holder with a suction port on the side, and Ag/AgCl was used to make contact with the electrode solution. To ensure that the recording was stable and continuous, we measured various types of ionic currents in the whole-cell mode of the standard patch-clamp technique by using either an Axoclamp-2B, an Axopatch-200B (Molecular Devices, Sunnyvale, CA, USA), or an RK-400 amplifier (Bio-Logic, Claix, France) [44,45]. Consistent with previous observations [46], the formation of a bleb of membrane lipids in the electrode tip (based on a microscopic observation of the seal formation) was also noticed. The cell membrane capacitance was measured at 13–24 pF (16.7 ± 2.6 pF, *n* = 27), whereas the series resistance under whole-cell current recordings was 64 ± 5 MΩ (*n* = 26). No series compensation was made during the measurements. The compounds that we tested were either applied through perfusion or added to the bath in order to achieve the final concentration indicated.

The signals were monitored at 10 kHz and stored online in an ASUS VivoBook Flip 14 laptop computer (TP412FA-0131A10210U; ASUS, Tainan, Taiwan) equipped with a Digidata 1440A interface (Molecular Devices). Parts of the experiments were also monitored on a Hantek-6022BC oscilloscope (Qingdao, Shangdong, China). During the measurements performed with analog-to-digital and digital-to-analog conversions, the Digidata-1440A device was controlled by means of pCLAMP 10.7 software (Molecular Devices) run on Microsoft Windows 10 (Redmond, WA, USA). Through the digital-to-analog conversion, pCLAMP-created voltage-clamped protocols with varying rectangular and ramp waveforms were specifically designed and deemed suitable for determining the steady-state and instantaneous relationships between the current and voltage (*I–V*) [45], as well as for studying the voltage-dependent hysteresis of specific ionic currents (e.g., the *I*_Na(P)_ and *I*_Ca,L_).

To calculate the percentage inhibition of ZO of the magnitude of the *I*_Na_ (the transient and late components) and the peak *I*_Ca,L_, the examined GH_3_ cells were depolarized by using a 40 msec short command pulse from a holding potential ranging between −80 mV and −10 mV and a 500 msec voltage pulse ranging between −50 mV and +10 mV, respectively. The amplitude of the currents during cell exposure to ZO was compared with the control conditions (i.e., when ZO was not present). In an effort to optimize the parameter values (i.e., IC_50_, n_H_ and *E*_max_), the ZO concentration required to inhibit 50% of the current magnitude was fitted to a logistic equation (i.e., a modified form of the Hill equation) using the least-squares method:

$$y=\frac{E_{max}}{1+\frac{IC_{50}^{n_{H}}}{{\left[C\right]}^{n_{H}}}},$$

where y is the percentage inhibition of the current amplitude; [*C*] is the ZO concentration; IC_50_ is the ZO concentration at which the half-maximal inhibition of the *I*_Na_ (the transient and late components) and the *I*_Ca,L_ was achieved; n_H_ is the Hill coefficient; and *E*_max_ is the ZO-induced maximal inhibition of the *I*_Na_ (the peak and late components) and the *I*_Ca,L_.

To estimate the fast and slow components of the *I*_Na_ inactivation time course, the trajectory of current traces with and without the addition of different ZO concentrations was appropriately fitted to the following equation:

$$I\left(t\right)=I_{\max\left(F\right)}\times \left[exp\left\{-\frac{1}{\tau_{inact\left(F\right)}}\right\}\right]+I_{\max\left(S\right)}\times \left[exp\left(-\frac{1}{\tau_{inact\left(S\right)}}\right)\right]$$

where *τ_max(F)_* and *τ_max(S)_* represent the time constant for the fast and slow components of the *I*_Na_ inactivation elicited by rapid depolarizing pulses; and *I*_max(F)_ and *I*_max(S)_ are the fast and slow components at the peak amplitude of the *I*_Na_, respectively.

Curve fitting to the experimental data sets was performed with either an iterative linear or non-linear regression analysis with goodness-of-fit measures. The values were expressed as means ± standard error of mean (SEM), with sample sizes (*n*) showing the cell number from which the experimental observations were collected, unless stated otherwise. The data distribution was found to satisfy the tests (frequency distribution and the Kolmogorov–Smirnov test) for normality. For comparisons between the two groups, statistical significance was evaluated using a Student’s *t*-test (paired or unpaired), whereas comparisons between more than two groups were made using an analysis of variance followed by the post-hoc Fisher’s least-significance difference method for multiple comparisons. A *p* value of 0.05 or less was considered significant, unless stated otherwise.

## 5. Conclusions

The experimental results specified herein suggest that the ZO-mediated perturbation of the amplitude, gating kinetics, and hysteresis of ionic currents tends to occur upstream of its effect on the production of reactive oxygen species, and that it is involved in moderating important functional activities occurring in neuronal cells.

## Data Availability

Data are available upon request from the corresponding authors.
